# Chronic cerebral hypoperfusion causes decrease of O-GlcNAcylation, hyperphosphorylation of tau and behavioral deficits in mice

**DOI:** 10.3389/fnagi.2014.00010

**Published:** 2014-02-10

**Authors:** Yang Zhao, Jin-hua Gu, Chun-ling Dai, Qun Liu, Khalid Iqbal, Fei Liu, Cheng-Xin Gong

**Affiliations:** ^1^Department of Neurology, The First Hospital of Jilin University, ChangchunJilin, China; ^2^Department of Neurochemistry, New York State Institute for Basic Research in Developmental DisabilitiesStaten Island, NY, USA; ^3^Department of Pharmacology, Medical School, Nantong UniversityNantong, Jiangsu, China

**Keywords:** chronic cerebral hypoperfusion, Alzheimer’s disease, cognitive impairment, O-GlcNAcylation, tau phosphorylation, synaptic plasticity markers, brain insulin signaling, neurodegeneration

## Abstract

Chronic cerebral hypoperfusion (CCH) is one of the causes of vascular dementia (VaD) and is also an etiological factor for Alzheimer’s disease (AD). However, how CCH causes cognitive impairment and contributes to Alzheimer’s pathology is poorly understood. Here we produced a mouse model of CCH by unilateral common carotid artery occlusion (UCCAO) and studied the behavioral changes and brain abnormalities in mice 2.5 months after UCCAO. We found that CCH caused significant short-term memory deficits and mild long-term spatial memory impairment, as well as decreased level of protein O-GlcNAcylation, increased level of tau phosphorylation, dysregulated synaptic proteins and insulin signaling, and selective neurodegeneration in the brain. These findings provide mechanistic insight into the effects of CCH on memory and cognition and the likely link between AD and VaD.

## INTRODUCTION

Vascular dementia (VaD) is the second most common form of dementia after Alzheimer’s disease (AD) in older adults (Battistin and Cagnin, 2010), accounting for ~20% of all dementia cases worldwide (Dubois and Hébert, 2001). VaD is defined as the loss of cognitive function to a degree that interferes daily living activities caused by problems in blood supply to the brain, which injures brain regions important for memory, cognition, and behavior (Roman, 2002). Chronic cerebral hypoperfusion (CCH) is a major cause of VaD and can result from disorders that affect cerebral vascular system, including hypertension, diabetes, generalized atherosclerosis, and smoking (Meyer et al., 2000; Valerio Romanini et al., 2013). Individuals with CCH usually have cognitive deficits to various degrees. The important role of CCH in dementia has already immerged to the front edge of neurology research. Studies in the last decade have suggested that CCH might promote neurodegeneration through neuronal energy failure, production of reactive oxygen species and proinflammatory cytokines by activated microglial cells that, in turn, damage the neuronal cells, and contribute to white matter lesions (Kitagawa et al., 2005; Farkas et al., 2007; Adibhatla and Hatcher, 2008; Urabe, 2012; Bang et al., 2013).

Cerebral vascular abnormalities are also an important contributing factor for AD (Kalaria et al., 2012; Leszek et al., 2012; Sato and Morishita, 2013; Sudduth et al., 2013). It is estimated that as many as 40% of AD patients actually have a mixed dementia of AD and VaD (Kammoun et al., 2000). AD is characterized by chronic and progressive neurodegeneration leading to progressive cognitive dysfunction and eventually to death of the patients. AD in most cases is sporadic and is probably caused by multiple factors. AD is histopathologically characterized by the presence of both intraneuronal neurofibrillary tangles (NTFs) and extracellular senile plaques together with neurofibrillary degeneration in the brain. NFTs are predominantly comprised of aggregated abnormally hyperphosphorylated tau (Grundke-Iqbal et al., 1986a,b), whereas senile plaques are made of aggregated amyloid-β (Aβ) peptides. We have previously found that impaired brain glucose metabolism in AD probably lead to abnormal hyperphosphorylation of tau and neurofibrillary degeneration via down-regulation of tau O-GlcNAcylation, a post-translational modification of proteins with β-N-acetylglucosamine (GlcNAc; Liu et al., 2004, 2009). Thus, we postulate that CCH might lead to chronic neurodegeneration and cognitive impairment in AD and VaD through reduction of cerebral glucose metabolism and consequently down-regulation of O-GlcNAcylation and hyperphosphorylation of tau. The present study was aimed to test this hypothesis in a mouse model of CCH.

A common rodent model of CCH is produced by unilateral common carotid artery occlusion (UCCAO; Levine, 1960; Rice et al., 1981). A 35–55% decrease of cerebrocortical perfusion is reported in the ipsilateral hemisphere in mice 28 days after UCCAO surgery (Kitagawa et al., 2005). To investigate the role of CCH in AD-related brain abnormalities and cognitive impairment, we performed UCCAO in adult mice and then studied general and memory behavior, O-GlcNAcylation, tau phosphorylation, synaptic proteins, hexosamine biosynthetic pathway (HBP) and insulin signaling. We found that CCH caused anxiety and deficits of motor functions and spatial memory, led to a decrease in O-GlcNAcylation and an increase in tau phosphorylation, dysregulated synaptic proteins, and resulted in alterations of the HBP and insulin signaling pathway in the mouse brains.

## MATERIALS AND METHODS

### ANTIBODIES AND REAGENTS

Primary antibodies used in this study are listed in **Table 1**. Peroxidase-conjugated anti-mouse and anti-rabbit IgG were obtained from Jackson ImmunoResearch Laboratories (West Grove, PA, USA). The enhanced chemiluminescence (ECL) kit was from Pierce (Rockford, IL, USA). Avidin-biotin complex (ABC) staining system was from Santa Cruz Biotechnology (Santa Cruz, CA, USA). Other chemicals were from Sigma (St. Louis, MO, USA).

**Table 1:** Primary antibodies used in this study.

Antibody	Type	Specificity	Phosphorylatio*n* sites	Source/Reference
HIF-1β	Mono-	HIF-1β		Abcam, Cambridge, MA, USA
TIGAR	Poly-	TIGAR		Abcam
GLUT1	Poly-	GLUT1		Millipore, Temecula, CA, USA
GLUT2	Poly-	GLUT2		Milipore, Billerica, MA, USA
GLUT3	Poly-	GLUT3		Santa Cruz Biotech., Santa Cruz, CA, USA
OGT	Poly-	OGT		Sigma, St. Lious, MO, USA
OGA	Poly-	OGA		Crawford et al. (2008)
CTD110.6	Mono-	O-GlcNAc		Covance, Emeryville, CA, USA
RL2	Mono-	O-GlcNAc		Affinity Bioreagents, Golden, CO, USA
pS199	Poly-	P-tau	Ser199	Invitrogen, Grand Island, NY, USA
pT205	Poly-	P-tau	Thr205	Invitrogen
pT212	Poly-	P-tau	Thr212	Invitrogen
pS214	Poly-	P-tau	Ser214	Invitrogen
pT231	Poly-	P-tau	Thr231	Invitrogen
pS262	Poly-	P-tau	Ser262	Invitrogen
pS396	Poly-	P-tau	Ser396	Invitrogen
pS404	Poly-	P-tau	Ser404	Invitrogen
R134d	Poly-	Tau		Tatebayashi et al. (1999)
Synapsin1	Poly-	Synapsin 1		Santa Cruz Biotech.
Synaptophysin	Mono-	Synaptophysin		Millipore
PSD95	Mono-	PSD95		Cell Signaling Tech, Danvers, MA, USA
GFAT2	Poly-	GFAT2		Santa Cruz Biotech.
IRβ	Poly-	IRβ		Cell signaling Technology, Danvers, MA, USA
IGF-1Rβ	Poly-	IGF-1Rβ		Cell signaling Technology
P-IRβ/IGF-1Rβ	Mono-	P-IRβ/IGF-1Rβ	Tyr1150/1151(IRβ), Tyr1135/1136 (IGF-1Rβ)	Cell signaling Technology
IRS1	Poly-	IRS1		Cell Signaling Technology
IRS1 pS307	Poly-	P-IRS1	Ser307	Cell Signaling Technology
PI3K p85	Poly-	PI3K (p85)		Cell Signaling Technology
P-PI3K p85	Poly-	P-PI3K (p85)	Tyr458/Tyr199	Cell Signaling Technology
PDK1	Poly-	PDK1		Cell Signaling Technology
PDK1 pS241	Poly-	P-PDK1	Ser241	Cell Signaling Technology
AKT	Poly-	AKT		Cell Signaling Technology
AKT pS473	Poly-	P-AKT	Ser473	Cell Signaling Technology
GSK-3β	Poly-	GSK-3β		Cell Signaling Technology
GSK-3β pS9	Poly-	P-GSK-3β	Ser9	Cell Signaling Technology
GAPDH	Poly-	GAPDH		Santa Cruz Biotech.

### ANIMALS AND UCCAO

Breeding pairs of mice (B6.129) were originally obtained from the Jackson Laboratory (New Harbor, ME, USA), and the mice were bred at our institutional animal colony. They were housed (4~5 animals per cage) with a 12/12 h light/dark cycle and with ad libitum access to food and water. The housing, breeding, and animal experiments were in accordance with the approved protocol from our Institutional Animal Care and Use Committee and with the PHS Policy on Human Care and Use of Laboratory animals (revised March 15, 2010).

**FIGURE 1:**
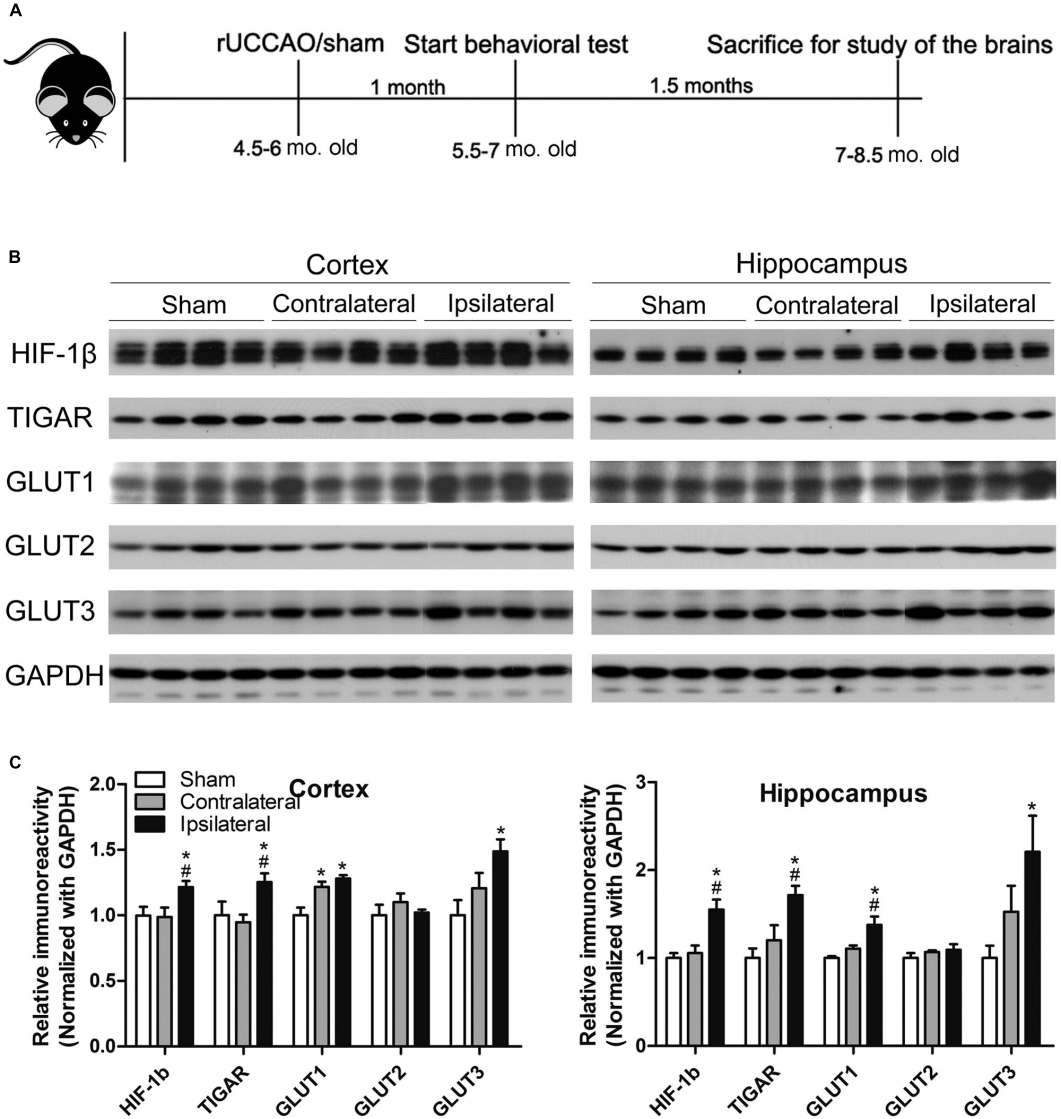
**Animal study design (A) and verification of the UCCAO model (B,C).** Homogenates of the cortices and hippocampi of mice 2.5 months after sham or UCCAO surgery were analyzed by Western blots developed with the indicated antibodies **(B)**. Each lane represents an individual mouse sample. The blots were then quantified densitometrically, and the data after normalization with the GAPDH level are presented as mean ± SEM (*n* = 6/group), where the values of the sham group are set as 1.0 **(C)**. *p < 0.05 vs. sham group; #*p* < 0.05 vs. contralateral group.

Male mice of 4.5–6 months old were randomly divided into two groups. The CCH group mice (*n* = 10) were subjected to unilateral (right side) common carotid artery (CCA) occlusion, and the control mice (*n* = 12) underwent the same procedure without vessel occlusion (sham surgery). The UCCAO was performed by permanent vessel double ligation under general anesthesia with 2.5% avertin. Briefly, a ventral midline incision was made to expose the right CCA. The artery was gently isolated from the carotid sheath and vagus nerve and the right CCA was doubly ligated with 9-0 silk suture (Ethicon^®^) just below the bifurcation. The midline incision was then closed carefully, and the animals were transferred back into their cages with free access to water and food. One month after the surgery, the mice were subjected to a battery of behavioral tests which lasted for 6 weeks (**Figure 1A**). After surgery, the body weight and food intake were monitored weekly. After completion of all behavioral tests, the mice were sacrificed by decapitation 2.5 months after the surgery, and the brains were removed immediately. The cerebral cortex and the hippocampus of the right hemispheres of the sham mice and of both hemispheres of the UCCAO mice were dissected, flash frozen in dry ice, and stored in –80°C for biochemical analyses. Some brains were fixed with 4% paraformaldehyde in 0.1 M PBS, followed by cryoprotection in 30 % sucrose. Coronal sections (40 μm thick) were cut on a freezing microtome. The sections were stored in glycol anti-freeze solution (ethylene glycol, glycerol, and 0.1 M PBS in a 3:3:4 ratio) at -20°C until immunohistochemical staining.

### BEHAVIORAL TESTS

All behavioral tests were performed between 8:00 a.m. and 5:00 p.m. Before the behavioral tests started, the mice were habituated by being handled by experimenters. Before each test, mice were transported to the test room (temperature was 23 ± 1°C) located inside of the animal colony to acclimatize for 1 h.

#### Open field

Exploratory activity, anxiety and locomotor activity were measured using the open field test. The apparatus was constructed of white painted plywood measured 50 cm × 50 cm as the square arena and 40 cm high PVC walls. A central square of 20 cm × 20 cm was surmounted by an automated camera-based video tracking system (ANY-Maze version 4.5 software; Stoelting Co., Wood Dale, IL, USA). Each mouse was placed individually in the arena and allowed to explore for 15 min, and the overall performance including distance traveled, distance in central area, central area entries and time spent in central area were recorded by the video tracking system. After the 15 min test, mice were returned in their home cages. Between test of each mouse, all urines and defecations were cleared from the field, followed by cleaning with 70% ethyl alcohol and air drying of the field.

#### Elevated plus maze

Elevated plus maze measures anxiety induced by open spaces, as well as height related anxiety (Walf and Frye, 2007). The apparatus was an elevated (80 cm high) maze consisting of four arms (30 cm × 5 cm each) connected by a common 5 cm × 5 cm central area making the shape of a plus sign. Two of the arms facing opposite were enclosed by 20 cm-high walls and were thus called closed arm (CA), whereas the other two facing arms were open and were thus called open arm (OA). Each mouse was placed onto the central area facing an OA and allowed to explore the maze for 8 min. The automatic video tracking system was used for collecting behavioral data including the number of CA entries, OA entries, and time spent in CA and OA. Between test of each mouse, the maze floor was cleaned as described above for the open field.**

#### Novel object recognition

Mice have a tendency to interact more with a novel object than with a familiar one. This tendency has been used by behavioral pharmacologists and neuroscientists to study learning and memory. A popular protocol for such research is the object recognition task (Bevins and Besheer, 2006). The test apparatus was the same one as used in the open field test described above. The procedure consisted of three phases: habituation phase, sample phase, and test phase. During habituation phase, each mouse was allowed to explore the field in the absence of objects for 15 min for four consecutive days in order to make the mice become familiar with the field. On the fifth day during the sample phase, two objects were placed symmetrically onto the arena. The mouse was placed at the mid-point of the wall opposite the sample objects with its body parallel to the side walls and its nose pointing away from the objects, allowed to freely explore for 5 min. Time spent exploring the objects was recorded. During the test phase, one of the two objects used in sample phase was randomly replaced by a novel one, the mouse was reintroduced to the arena for 5 min exploration after a 15 min delay. Another 5 min test session was performed 24 h later for evaluation of long-term memory retention (Ennaceur and Delacour, 1988). Between each mouse, any feces were cleared, and the arena and objects were cleaned with 70% ethyl alcohol. The video tracking system was used to collect behavioral performances automatically. Time spent exploring both the novel and the familiar objects was recorded (*T*_N_, T_F_). Object discrimination was evaluated by the recognition index (RI): RI = *T*_N_/(*T*_N_ +* T*_F_).

#### Accelerating Rotarod test

Motor coordination and motor learning of mice were assessed by using Rotarod test. The mouse was placed on the roller lane of the Rotarod (Panlab, LE8500, Spain) and started rotating the rod with a steady acceleration from 4 to 40 rpm over a 5 min period. The latency at which the mouse fell off the rod was recorded. Each mouse was given six trials with 10–15 min inter-trial intervals (ITIs).**

#### Forced swim test

Depression-like behaviors were evaluated through forced swim test. A transparent plastic beaker (17 cm in diameter × 24 cm high) containing water (23 ± 1°C) to a depth of 17 cm was used as the swimming apparatus. All mice were individually forced to swim for 6 min, and the duration of immobility was observed and measured during the final 4 min of the test. Immobility consists of the mouse floating in the water without struggling and only making movements necessary to keep its head above water surface (Slattery and Cryan, 2012).

#### Footprint test

Motor coordination and gait were assessed through footprint test using a 50 cm long and 8 cm wide runway (with 10 cm high walls). Before collecting the footprints, each mouse was trained for three or four runs to make sure it was able to run along the runway at a steady pace and in a straight line without wandering. To obtain footprints, the forepaws and hindpaws of the mice were respectively coated with red and blue non-toxic paints. To collect the footprints, a fresh sheet of pale white paper was placed on the floor of the runway for each run. The footprint patterns were analyzed for the following four step parameters (all measured in centimeters). (1) Stride length was measured as the average distance of forward movement between each stride. The left/right forelimb and left/right hindlimb stride were also measured separately. (2) Front/hind base width was measured as the average distance between left and right front/hind footprints, respectively. These values were determined by measuring the perpendicular distance of a given step to a line connecting its opposite preceding and proceeding steps. (3) Overlap between forepaw/hindpaw placements were measured as the distance between the front/hind footprints on each side. When the center of the hind footprint fell on top of the center of the preceding front footprint, a value of zero was recorded (Carter et al., 1999).

#### Morris water maze

Morris water maze (MWM) was used to evaluate spatial learning and memory of the mice (D’Hooge and De Deyn, 2001). The test was performed in a circular white pool (with a diameter of 180 cm and a height of 60 cm) filled with white dye tinted water and maintained at room temperature (20 ± 1°C). The maze was designated of two principal axes with each line bisecting the maze perpendicular to the other one to divide the maze into four equal quadrants. The end of each line demarcates four cardinal points: north (N), south (S), east (E), and west (W). A platform was positioned in the middle of one of the quadrants submerged 1 cm below water surface. Each mouse performed four trials per day for four consecutive days from semi-random start positions (**Table 2**) to find the hidden platform. Each trial was terminated as soon as the mouse climbed onto the hidden platform. If a mouse failed to find the platform within 90 s, it was gently guided to it. At the end of each trial, the mouse was left on the platform during a 20 s ITI, then dried and returned to its home cage. A 60 s probe test without platform was performed 24 h after the last trial. The swim path, swim distance (cm), escape latency (sec), swim speed (cm/sec), time spent in each quadrant (sec), distance traveled in each quadrant (cm), latency to enter the platform site zone (sec), and the number of platform site zone crossings were recorded through an automated tracking system (Smart video tracking system, Panlab; Havard Apparatus). A reversal test was performed on the day after the probe day of original test. For the reversal test, all conditions were the same as the original test except the platform was relocated to the center of the opposite quadrant against the original one.

**Table 2:** Mouse start positions in the Morris water maze.

Day	Trial 1	Trial 2	Trial 3	Trial 4
**Original tests**
1	S	NW	SE	W
2	NW	W	S	SE
3	W	SE	NW	S
4	SE	S	W	NW
Probe	NE			
*platform site location: SW*
**Reversal tests**
1	N	E	SW	NW
2	SE	N	NW	W
3	NW	SW	W	N
4	E	NW	N	SE
Probe	SW			

**platform site location: NE**

### WESTERN BLOT ANALYSIS

Mouse brain tissue was homogenized in pre-chilled buffer containing 50 mM Tris-HCl (pH 7.4), 50 mM GlcNAc (inhibitor of O-GlcNAcase that catalyzes the removal of O-GlcNAc from a protein), 20 μM UDP (inhibitor of O-GlcNAc transferase), 2.0 mM EGTA, 2 mM Na_3_VO_4_, 50 mM NaF, 0.5 mM AEBSF, 10 μg/ml aprotinin, 10 μg/ml leupeptin, and 4 μg/ml pepstatin A. Protein concentrations of the homogenates were determined by using Pierce^TM^ 660 nm Protein Assay kit (Thermo Fisher Scientific Inc.). The samples were resolved in 10% or 12.5% sodium dodecyl sulfate polyacrylamide gel electrophoresis (SDS-PAGE) and electro-transferred onto Immobilon-P membrane (Millipore, Bedford, MA, USA). The blots were then probed with primary antibody (**Table 1**) and developed with the corresponding horseradish peroxidase–conjugated secondary antibody and ECL kit (Pierce, Rockford, IL, USA). Densitometrical quantification of protein bands in Western blots were analyzed by using the Multi Gauge V3.0 software (Fuji Photo Film Co., Ltd).

### IMMUNO-DOT-BLOT ASSAY

The level of O-GlcNAcylation in the brain homogenates was determined by using a quantitative immuno-dot-blot assay (Liu et al., 2002) with modifications. Briefly, homogenate samples were first dissolved in Laemmli buffer and then diluted with the homogenizing buffer till the SDS concentration was 0.05% or lower. The diluted samples were dotted onto nitrocellulose membrane (Schleicher and Schuell, Keene, NH, USA) at 6 ul per grid of 6 × 6 mm in size. The blots were placed in a 37°C oven for 0.5–1 h to allow the protein binding to the membrane. After the membrane was blocked with TBST (10 mM Tris-HCl, pH7.5, 150 mM NaCl, 0.3% Tween-20) for 1 h, the blots were overlaid with a mixture of antibodies RL-2 and CTD110.6 and then incubated at 4°C overnight. After washing with TBST, the blots were processed as described above for Western blots.

### IMMUNOHISTOCHEMICAL STAINING

Floating sagittal sections were incubated at room temperature in 0.3% H_2_O_2_ for 30 min and then in 0.3% Triton X-100 for 15 min, washed in PBS, and blocked in a solution containing 5% normal goat serum and 0.1% Triton X-100 for 30 min. Sections were then incubated at 4°C with primary antibody overnight, followed by development with biotinylated secondary antibody and avidin/biotinylated horseradish peroxidase (Santa Cruz Biotechnology). The sections were stained with peroxidase substrate and then mounted on microscope slides (Brain Research Laboratories, Newton, MA, USA), dehydrated, and covered with coverslips.

### FLUORO-JADE STAINING

Floating sagittal sections were mounted on gelatin coated slide, dried at room temperature overnight, and then rinsed in distilled water for 1 min. After incubation in freshly prepared 0.06% potassium permanganate for 10 min and washes with distilled water, sections were stained with 0.0004% Fluoro-Jade B in 0.09% acetic acid for 10 min. After washes again, the sections were further stained with DAPI. Stained sections were observed under a fluorescence microscope (PCM2000, Nikon, Melville, NY, USA), and positive cells were counted under 20 × magnification by the use of Image J software (National Institutes of Health).

### STATISTICAL ANALYSIS

For behavioral tests, data were analyzed by two-way ANOVA or unpaired two-tailed *t*-test using Graphpad Prism 5s. For biochemical analyses, data were analyzed by one-way ANOVA followed by Tukey’s *post hoc* tests or unpaired two-tailed *t*-tests, using Graphpad Prism 5. All data are presented as means ± SEM, and *p *< 0.05 was considered statistically significant.

## RESULTS

### VERIFICATION OF THE CCH MODEL

Previous studies have demonstrated that UCCAO does not produce histologically detectable cerebral ischemia or neuronal cell death (Kitagawa et al., 2005; Lee et al., 2011; Pimentel-Coelho et al., 2013). Thus, we first verified the success of UCCAO by detecting the levels of hypoxia inducible factor-1β (HIF-1β), TP53-induced glycolysis and apoptosis regulator (TIGAR) and glucose transporters (GLUTs) 1 and 3, because the expression of these proteins increases during cerebral or cardiac ischemia (Watanabe et al., 2009; Kimata et al., 2010; Chan et al., 2011; Iwabuchi and Kawahara, 2011; Yan et al., 2011; Yuan et al., 2011; Hoshino et al., 2012; Wang et al., 2012a,b). As expected, we found significant increases in the levels of these proteins in both the cerebral cortex and the hippocampus of the ipsilateral side of the UCCAO mice (**Figures 1B,C**). A trend of increase in GLUT1 and GLUT3 was also seen in the contralateral side, and this increase reached statistical significance for cerebrocortical GLUT1 (**Figure 1C**), which might suggest that there was mild chronic hypoperfusion at the contralateral hemisphere in the mice 2.5 months after UCCAO. As a negative control, the level of GLUT2 was not altered after UCCAO (**Figures 1B,C**). These results showed the success of CCH in the brains of mice after UCCAO.

### CCH CAUSES ANXIETY AND DEFICITS OF MOTOR FUNCTIONS

**FIGURE 2:**
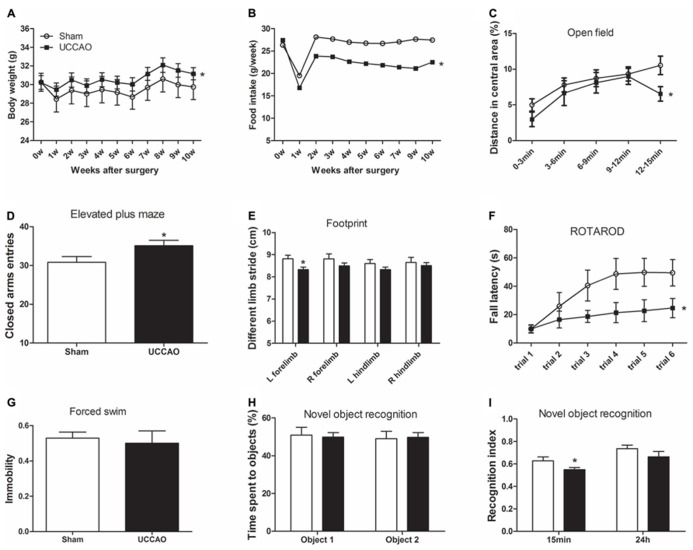
**General behavior and short-term memory of mice with CCH.** The body weight and food intake were monitored once a week after sham or UCCAO surgery **(A,B)**. Spontaneous locomotor and exploratory activity was assessed in an open field **(C)**. Anxiety-like behaviors were evaluated in an elevated plus maze, and the CAs entries are shown **(D)**. Gait was evaluated using footprint test, and different limb strides are shown **(E)**. Motor coordination and balance were evaluated using accelerating Rotarod, and the fall latency of each trial is shown **(F)**. Depression was evaluated using forced swim test, and last 4 min immobility out of total 6 min is shown **(G)**. One-trial object recognition task was carried out in an open field. Time spent exploring two identical objects during sample phase are shown as percentage of object exploring time **(H)**. Object discrimination during test phase is presented by the recognition index (time exploring the novel object/total time for exploring) **(I)**. Data are reported as mean ± SEM.*p < 0.05 vs. sham mice.

To investigate the effects of CCH on the mouse behavior, we first monitored general conditions of the mice, including body weight and food intake after sham or CCH surgery. We found that the body weights of both groups decreased one week after the surgery (**Figure 2A**), indicating the consequence of stress from the surgery itself on the mice. The body weight recovered a week later, but the CCH mice constantly had a significantly higher body weights (5–6% higher) than the sham mice. Interestingly, the CCH mice had a much lower food intake (~20% lower) than the sham mice after the marked decline during the first week after the surgery (**Figure 2B**). The surprising opposite changes of the body weight and food intake of the CCH mice suggest that CCH might have caused decreased metabolism and/or activity in mice.

We then assessed the general behaviors including exploratory activity, anxiety, and locomotor activity of the mice. By testing the mice in an open field, we found that the CCH mice traveled a shorter distance (**Figure 2C**) and spent less time (data not shown) in the central area of the open field than the sham mice. In an elevated plus maze for measuring open space- and height-induced anxiety, we found that the CCH mice entered the CAs more frequently (**Figure 2D**). Together, these results suggest an increased level of anxiety and a decreased level of spontaneous exploratory activity of the CCH mice, the latter of which is consistent with the increased body weights and decreased food intake of the CCH mice observed above.

To study whether UCCAO changes the gait and motor coordination of the mice, we carried out foot print test and Rotarod test. We found that the left forelimb stride of the CCH mice was slightly shorter than that of the sham mice as measured in the footprint test (**Figure 2E**). However, the decrease in the left forelimb stride of the CCH mice was not statistically significant as compared to the right forelimb stride of the same mice. The Rotarod test showed marked decrease of the fall latency of the CCH mice as compared to the sham group (**Figure 2F**). These results indicate that the CCH mice might have a slight decrease in motor activity of left side (contralateral side) and a pronounced decrease in motor coordination and motor learning. However, forced swim test did not show any detectable abnormality in swimming, nor the time spent immobile in the CCH mice (**Figure 2G**), indicating that the CCH mice were not under a depressive state.

### CCH LEADS TO MILD IMPAIRMENT IN MEMORY

The non-spatial working memory related to frontal subcortical circuits of the mice was assessed by using object recognition test (Sarti et al., 2002) with 15 min and 24 h intervals between the sample phase and the test phase. During the sample phase, all mice spent the same amount of time in exploring both objects (**Figure 2H**). However, the CCH mice spent less time exploring the novel object than the sham mice when tested both 15 min and 24 h after the sample phase, although the decrease only at the 15 min time point reached the statistical significance (**Figure 2I**). These results indicate a short-term memory deficit in the CCH mice.

**FIGURE 3:**
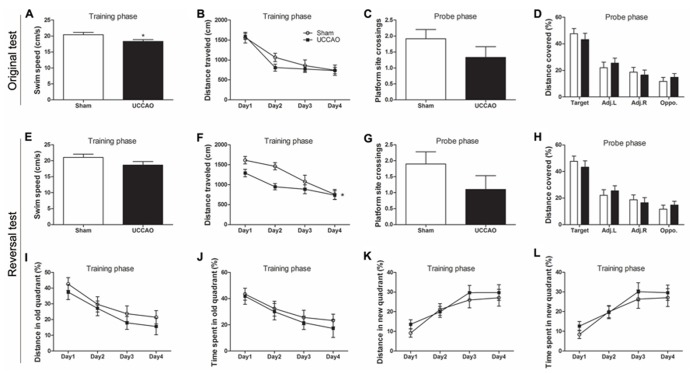
**Morris water maze tests of mice with CCH.** Spatial memory of the mice was tested in the Morris water maze test **(A–D)** and the consecutive reversal test **(E–L)**. The average swim speed in the training phase **(A,E)**, the distance traveled to the hidden platform during training **(B,F)**, the number of the platform site crossings during the probe trial **(C,G)**, the percentage of distance traveled in the target, adjacent left (Adj.L), adjacent right (Adj.R), and opposite (Opp) quadrants during probe trial **(D,H)** and the gradual reduction in percent time/distance in the quadrant of previous platform and the gradual shift to the new quadrant **(I–L)** are shown. Data are reported as mean ± SEM.*p < 0.05 vs. sham mice.

We also employed MWM to investigate the long-term spatial reference learning and memory of these mice. We observed a slight but significant decrease in swim speed of the CCH mice during the training phase (**Figure 3A**), which is consistent with the deficit in locomotor activity of these mice, as detected above. During the training phase in the water maze, both groups showed proper learning as evidenced by continuous decrease in the distance traveled (**Figure 3B**) and the latency (data not shown) to locate the hidden escape platform. Retrieval of spatial memory was more specifically tested with the probe trial performed 24 h after the last training trial. Memory performance of the mice was determined by the number of the former platform site crossings and the percentage of time/distance animals spent/traveled in the target quadrant. We found that the CCH mice showed a decrease in the number of platform crossings (**Figure 3C**), a slight increase of the latency to first crossing (data not shown), and slightly less preference toward the target quadrant (**Figure 3D**). However, none of these changes observed in the CCH mice reached a statistical difference. These results suggest that the CCH mice did not have a deficit in spatial memory or the tests and/or the sample size we used did not have sufficient power to detect the mild deficit of spatial memory in the CCH mice.

In an attempt to increase the sensitivity of the spatial memory test, we further carried out five days’ reversal test in the MWM starting on the next day following the probe test of the original test. For the reversal test, the escape platform was relocated to the quadrant opposite to the original quadrant. We observed that the CCH mice again had a lower swim speed (**Figure 3E**), a marked decrease in the mean platform site crossing (**Figure 3G**) and slightly less preference toward the target quadrant (**Figure 3H**) at the probe trial of the reversal test. However, these changes did not reach statistical significance, apparently due to large scattering of the data. The training curve of the reversal test indicated that the CCH mice traveled a shorter distance (**Figure 3F**) and had a shorter latency (data not shown) to locate the new hidden escape platform during the first three days of the training phase. To learn if this was because the CCH mice remembered the previous escape less well than the sham mice, we analyzed the distance traveled and the time spent in the quadrant where the previous old hidden escape platform was located. We found that the CCH mice traveled a shorter distance (**Figure 3I**) and spent shorter time (**Figure 3J**) in that quadrant than the sham mice, suggesting that the apparently better performance of the CCH mice during the first three days’ training in the reversal water maze, as seen in **Figure 3F**, was actually because these mice remembered the old platform location less well than the sham mice. The CCH and the sham mice showed no difference in the distance traveled (**Figure 3K**) and the time spent (**Figure 3L**) in the quadrant where the new escape platform was located during the training phase of the reversal water maze test, again suggesting no learning deficit of the CCH mice. Taken together, these results suggest that the CCH mice had no impairment in learning, but had a slight impairment in long-term spatial memory.

### CCH LEADS TO DECREASED O-GlcNAcylation AND INCREASED TAU PHOSPHORYLATION

**FIGURE 4:**
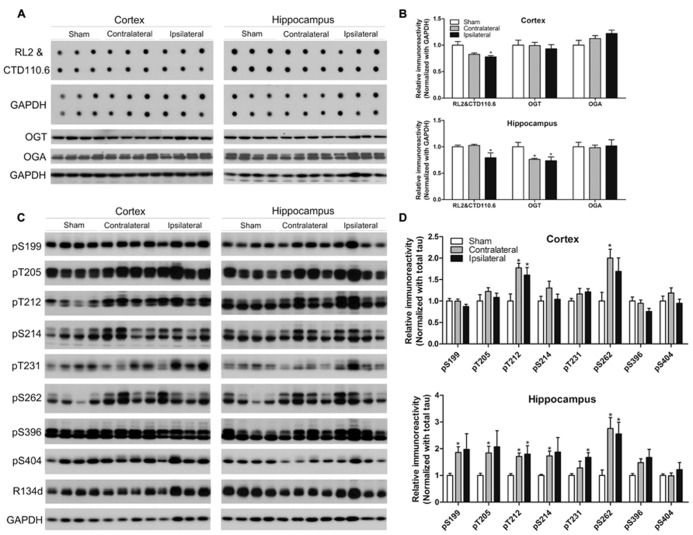
**Effects of CCH on O-GlcNAcylation and tau phosphorylation.** Homogenates of the cortices and hippocampi of mice 2.5 months after sham or UCCAO surgery were analyzed by immuno-dot blots developed with a mixture of monoclonal antibodies RL2 and CTD110.6 against O-GlcNAcylated proteins and Western blots developed with the indicated antibodies **(A,C)**. The blots were then quantified densitometrically, and the data after normalization with the GAPDH **(B)** or R134d for total tau level **(D)** are presented as mean ± SEM (*n* = 6/group), where the values of the sham group are set as 1.0.*p < 0.05 vs. sham group.

Because CCH is expected to reduce brain glucose metabolism and O-GlcNAcylation is a sensor of intracellular glucose metabolism (Zachara and Hart, 2004) and regulates tau phosphorylation negatively (Liu et al., 2004, 2009; Li et al., 2006), we investigated protein O-GlcNAcylation and its regulating enzymes O-GlcNAc transferase (OGT, which adds GlcNAc groups to proteins) and O-GlcNAcase (OGA, which removes GlcNAc from proteins), as well as tau phosphorylation. We found a significant decrease in the O-GlcNAcylation level in both the cortex and the hippocampus of the ipsilateral side of the CCH mice, as determined by immuno-dot blots developed with a mixture of two monoclonal antibodies both against various O-GlcNAcylated proteins (**Figures 4A,B**). To uncover the underlying mechanism for the change of O-GlcNAcylation, we determined OGT and OGA levels by Western blots and observed significant decrease in the OGT level in the hippocampus of the CCH mice (**Figures 4A,B**), which might partially explain the decreased O-GlcNAcylation.

Site-specific tau phosphorylation in the mouse brains was determined by Western blot developed with several phosphorylation-dependent and site-specific tau antibodies, and the total tau by a polyclonal pan-tau antibody R134d. We found that tau phosphorylation increased markedly at most of the phosphorylation sites studied in the hippocampus and at Thr212 and Ser262 of the cortex of the CCH mice (**Figures 4C,D**), which is consistent with the decrease of O-GlcNAcylation. Surprisingly, the increased tau phosphorylation was also seen at the contralateral side, where O-GlcNAcylation was not found to be decreased, of the UCCAO mice, suggesting that additional mechanism might be involved in tau phosphorylation at the contralateral side of the UCCAO mice.

### CCH DYSREGULATES SYNAPTIC PROTEINS

**FIGURE 5:**
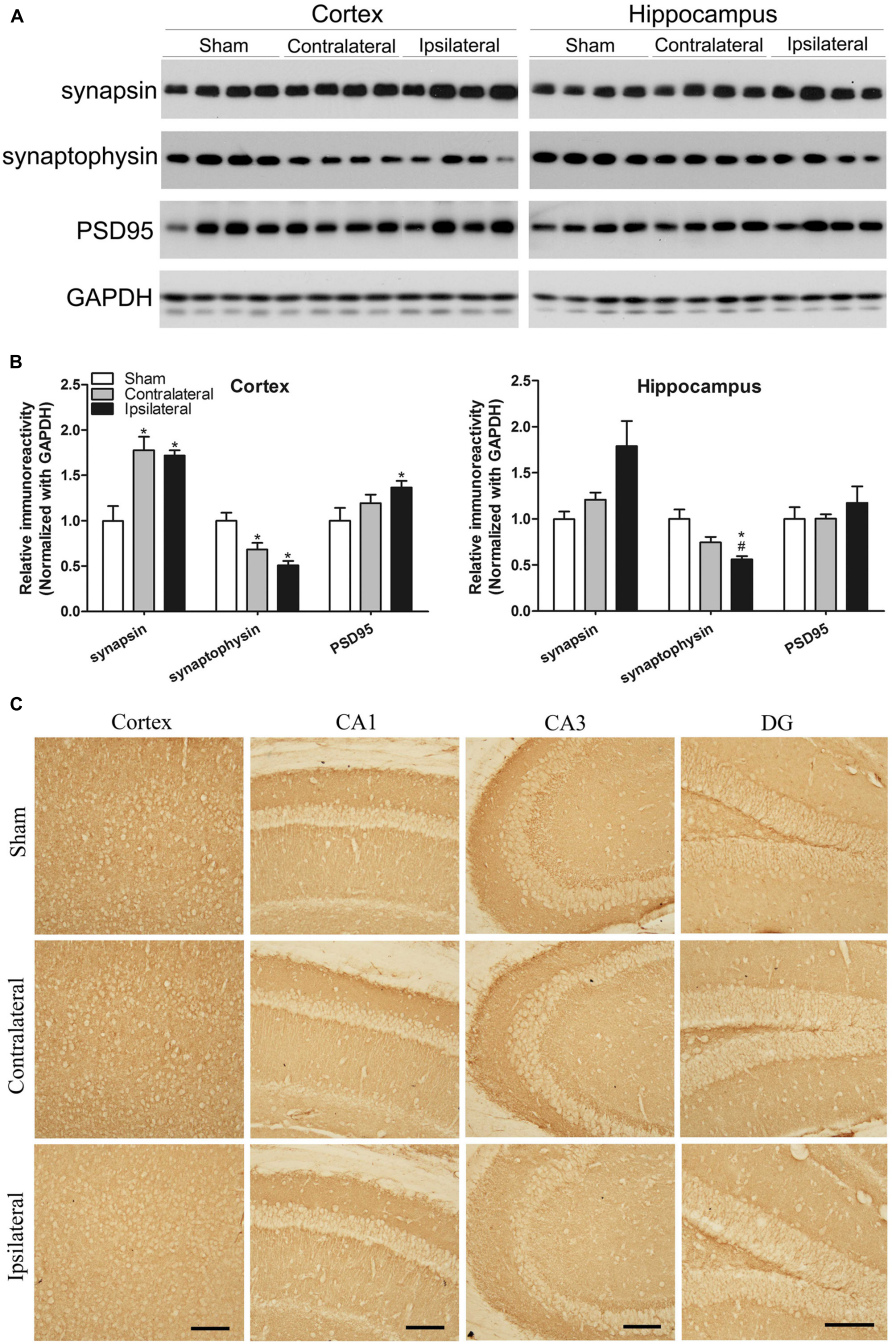
**Effects of CCH on synaptic proteins.** Homogenates of the cortices and hippocampi of mice 2.5 months after sham or UCCAO surgery were analyzed by Western blots developed with the indicated antibodies **(A)**. The blots were then quantified densitometrically, and the data after normalization with the GAPDH level are presented as mean ± SEM (*n* = 6/group), where the values of the sham group are set as 1.0 **(B)**.*p < 0.05 vs. sham group. #*p* < 0.05 vs. contralateral group. The coronal brain sections were immunostained with antibody against synaptophysin **(C)**. CA, Cornu Ammonis; DG, dentate gyrus; Scale bar: 100 μm.

Synapses are the structural basis of memory and cognition, which depends on synaptic plasticity that is in turn regulated by modulation of neurotransmitter release from the pre-synaptic site and of the number, types and properties of neurotransmitter receptors at the post-synaptic site. To understand the molecular basis of the mild memory impairment we detected in the CCH mice, we determined the levels of major pre- and post-synaptic proteins in the mouse brains. We found a dramatic increase in the level of synapsin but marked decrease in synaptophysin, both of which are commonly used as pre-synaptic markers, in both the cerebral cortex and the hippocampus of the CCH mice (**Figures 5A,B**). The level of post-synaptic density 95 (PSD95), a post-synaptic marker, was also found to be increased in the cerebral cortex of the CCH mice. These results indicate a dysregulation of synaptic proteins in the brains of the CCH mice. Interestingly, the contralateral side of the CCH mice tended to have the similar dysregulation of synaptic proteins, implicating that milder but similar changes might occur at the contralateral side of the CCH mice.

We also carried out immunohistochemical studies of synaptophysin in the mouse brains. The decreased immunostaining was seen throughout the cerebral cortex and various sectors of the hippocampus, more apparent in the ipsilateral side (**Figure 5C**). These results are consistent with the results from Western blots.

### CCH AFFECTS THE HBP AND INSULIN SIGNALING PATHWAY IN THE BRAIN

**FIGURE 6:**
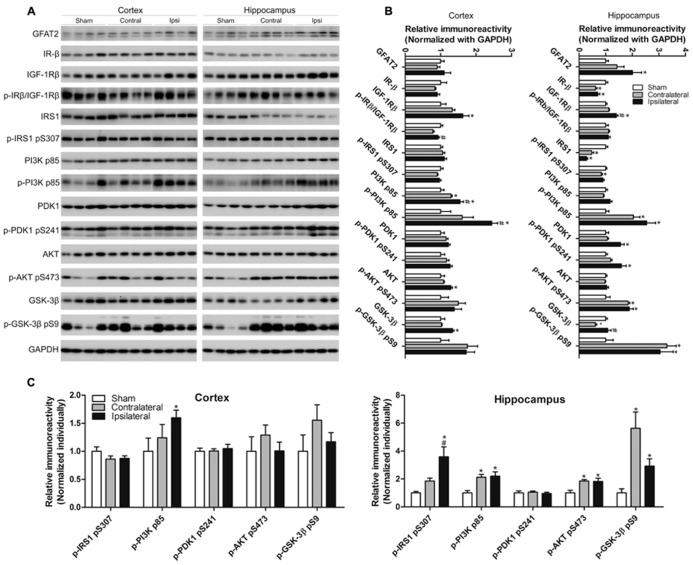
**Effects of CCH on the GFAT2 and the insulin signaling pathway.** Homogenates of the cortices and hippocampi of mice 2.5 months after sham or UCCAO surgery were analyzed by Western blots developed with the indicated antibodies **(A)**. The blots were then quantified densitometrically, and the data after normalization with the GAPDH **(B)** or the corresponding protein level **(C)** are presented as mean ± SEM (*n* = 6/group), where the values of the sham group are set as 1.0; *p < 0.05 vs. sham group. #*p* < 0.05 vs. contralateral group.

A small fraction of intracellular glucose is metabolized into uridine diphosphate-N-acetylglucosamine (UDP-GlcNAc), the donor of protein O-GlcNAcylation, through the HBP, and OGT activity is dependent on intracellular UDP-GlcNAc level (Hanover et al., 2010). The rate limiting enzyme of the HBP is L-glutamine:fructose-6-phosphate amidotransferase (GFAT). As we had observed a decreased level of O-GlcNAcylation after CCH, we further investigated the level of GFAT2, the major isoform of GFAT in the mammalian brain, and found that the GFAT2 level was increased in the ipsilateral hippocampus, but not in the cerebral cortex, of the CCH mice (**Figures 6A,B**).

Glucose metabolism as well as neural plasticity and memory are modulated by insulin signaling (Gerozissis, 2008; Cardoso et al., 2009), which is deregulated in AD brain (Liu et al., 2011). The classical insulin signaling pathway includes insulin receptor (IR), insulin-like growth factor-1 receptor (IGF-1R), insulin receptor substrate-1 (IRS-1), phosphatidylinositide 3-kinases (PI3K), 3-phosphoinositide-dependent protein kinase-1 (PDK1), protein kinase B (AKT), and glycogen synthase kinase-3 (GSK-3). This signaling is activated through the phosphorylation of each component by its upstream component of the signaling cascade. We therefore studied the brain insulin signaling by determining the level and activation status of each component of the insulin signaling pathway in the brain by quantitative western blots. The activation status was estimated by determining the level of phosphorylation, which reflects the enzymatic activity, with the phosphorylation-dependent antibodies. We observed changes of the level and/or phosphorylation of several components of the insulin signaling pathway in the ipsilateral side of the brains of UCCAO mice (**Figure 6**). These changes included increases in the levels of IGF-1Rβ, the p85 subunit of PI3K and its phosphorylation, PDK1 and its phosphorylation, and the phosphorylation of AKT and GSK-3β, as well as decreases in the levels of IR-β and IRS1 phosphorylation in the hippocampus. In case of the levels of IR-β, IRS1 and the phosphorylation of PI3K p85, AKT, and GSK-3β, the UCCAO-induced alterations were also seen in the contralateral side of the brain. The changes of insulin signaling in the cerebral cortex were somewhat similar but not identical to those seen in the hippocampus. Taken together, these results indicate that CCH results in alterations in insulin signaling.

### CCH LEADS TO NEURONAL DEGENERATION

**FIGURE 7:**
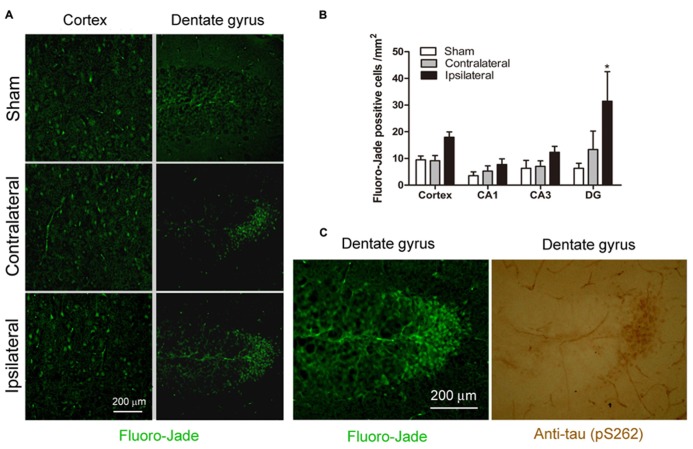
**Effects of CCH on neuronal degeneration.** Representative microphotographs of Fluoro-Jade stainining of cerebral cortex and dentate gyrus sections of mice 2.5 months after sham or UCCAO surgery **(A)**. The Fluoro-Jade positive neurons were then quantified by using the Image J software, and the data are presented as mean ± SEM (*n* = 6/group) **(B)**. Coexistence of hyperphosphorylated tau (detected using antibody against pS262) and Fluoro-Jade positive neurons was seen in adjacent sections **(C)**.*p < 0.05 vs. sham group.

To investigate whether CCH for 2.5 months causes neuronal degeneration, we stained the mouse brain frozen sections with Fluoro-Jade that labels degenerating neurons with green fluorescence. Overall, there were very few Fluoro-Jade positive neurons in the control mouse brain. We did not observe widespread Fluoro-Jade positive staining throughout the brain with CCH either. However, we found a slight increase in the number of Fluoro-Jade positive neurons in the ipsilateral cerebral cortex and hippocampus of the UCCAO brains, and this increase was most obviously seen in the granule cells of the crest of the dentate gyrus (**Figure 7A**). Quantification of the Fluoro-Jade positive neurons showed a significant increase in the ipsilateral side of cerebral cortex and dentate gyrus (**Figure 7B**). These results indicate that CCH for 2.5 months induces mild neurodegeneration and the dentate gyrus is the most sensitive area for CCH-induced neurodegeneration.

To investigate whether the CCH-induced chronic neurodegeneration is associated with tau hyperphosphorylation, we stained the adjacent brain sections with anti-pS262 that detects tau phosphorylated at Ser 262 and this immunoreactivity was increased dramatically in the CCH brains (**Figure 4**). We found a marked increase in anti-pS262 staining in neurons that are Fluoro-Jade positive in the dentate gyrus (**Figure 7C**). These observations suggest that the CCH-induced neuronal degeneration might be caused or associated with abnormal hyperphosphorylation of tau.

## DISCUSSION

The human brain constitutes only ~2% of the body’s mass, but it receives 15–20% of the body’s blood supply and accounts for up to 50% of the total body glucose utilization under the basal condition (Peters, 2011). Normal brain function requires a steady blood supply to maintain the stable energy state carrying out all of its cellular and molecular needs. When the blood supply to the brain becomes suboptimal for a long period of time, clinical implications involving chronic hypoperfusion and a variety of brain changes may occur. A series of experiments designed to mimic CCH have been achieved by permanent or reversible occlusion of two or three major vessels supplying the brain in rats. UCCAO is one of the approaches to produce CCH in mice and results in a 35–55% decrease of the ipsilateral cortical perfusion, as detected 28 days after the UCCAO surgery (Kitagawa et al., 2005). This model is thus ideal to investigate the contribution of CCH to cognitive deficits and AD pathology.

Glucose is the primary source of fuel for energy-demanding activities in mammalian brain. Together with oxygen, glucose is delivered by the circulation for the metabolic chores that keep brain cells healthy and functioning (Erecińska and Silver, 1989; Qutub and Hunt, 2005). The transport of glucose from blood into neurons requires GLUTs. Glucose is first transported from bloodstream into extracellular space through GLUT1, which is highly expressed in the endothelial cells of the blood–brain barrier, then further transported into neurons through neuronal GLUT3. As a response to the energy-demand following cerebral hypoperfusion, the expression and activity of these glucose transporters increase (Vannucci et al., 1997, 1998; Yu et al., 2008). This compensation is mainly mediated by the transcription factor HIF-1 that responds to hypoxia (Singh et al., 2012). TIGAR is a recently discovered enzyme that primarily functions as a regulator of glucose metabolism in the cell (Bensaad et al., 2006). Its expression is also elevated in responding to cardiac ischemia (Kimata et al., 2010). In the present study, we observed significant increases in the levels of HIF-1β, TIGAR, GLUT1, and GLUT3 in the ipsilateral side of the brain after UCCAO. These observations are consistent with the previous reports and help confirm the success of achieving CCH in the UCCAO mice.

In the present study by using the well-established UCCAO on 4.5–6 month old mice, we demonstrated that CCH caused anxiety, motor function deficits, and mild impairment of cognitive functions. These findings provide experimental evidence that CCH alone can lead to memory deficits. CCH for more than one month resulted in only mild cognitive deficits, indicating a large reserved capacity of the brain. Pimentel-Coelho et al. (2013) performed UCCAO on 4-month-old mice and did not find any cognitive deficits tested by using T-water maze task five weeks after UCCAO, but the same approach led to spatial learning impairments in APPswe/PS1 mice (Pimentel-Coelho et al., 2013). Our observations that mild cognitive impairment of the CCH mice was detectable only in the more sensitive reversal MWM, but not in the original MWM, are consistent with the above report. CCH’s initiation of cognitive impairment may be aggravated by other insults (such as overexpression of APPswe/PS1). A synergistic effects between overexpression of mutated APP and CCH on learning impairment in a mouse model, which over-expresses human APP bearing both the Swedish and Indiana mutations (APPSw/Ind-Tg mice), has been observed previously (Yamada et al., 2011). It is interesting for future studies to investigate the impact of CCH for a much longer time on cognitive function since many individuals could have cerebrovascular deficits years before the onset of memory loss in AD.

To investigate the possible molecular mechanisms that might underlie the CCH-induced cognitive impairment, we further investigated several AD-related molecules and pathways in the CCH mouse brain. Because O-GlcNAcylation is a sensor of intracellular glucose metabolism (Zachara and Hart, 2004) and is expected to be decreased under the hypoperfusion condition, we investigated protein O-GlcNAcylation. We found a significant decrease in the O-GlcNAcylation level in both the cortex and the hippocampus of the ipsilateral hemisphere of the UCCAO. These results indicate that the compensative increase in GLUT1 and GLUT3 might fail to compensate the intracellular glucose to the normal level. The reduction of O-GlcNAcylation level may result from a reduction of the intracellular level of UDP-GlcNAc, which is the donor for protein O-GlcNAcylation and is also the major activator of OGT activity, because UDP-GlcNAc is synthesized from glucose metabolism HBP. As we also found a reduction of OGT level in the hippocampus of the CCH brain, this reduction might also contribute to the decrease of O-GlcNAcylation in the hippocampus of the CCH mice.

It has been demonstrated that abnormal hyperphosphorylation of tau is crucial to neurodegeneration in AD and probably also in other tauopathies (Gong and Iqbal, 2008; Iqbal et al., 2013). Tau hyperphosphorylation can be promoted by several factors. One of these factors is decreased brain glucose metabolism through down-regulation of tau O-GlcNAcylation because the latter regulates tau phosphorylation inversely (Liu et al., 2004, 2009; Li et al., 2006). We therefore studied site-specific tau phosphorylation and found elevated tau phosphorylation at two sites (Thr212 and Ser262) in the cerebral cortex and six sites (Ser199, Thr205, Thr212, Ser214, Thr231, and Ser262) among the eight phosphorylation sites studied in the hippocampus in mice after CCH. Elevated tau phosphorylation at more sites in the hippocampus than in the cortex suggests that the hippocampus is more vulnerable to CCH-induced tau hyperphosphorylation. Higher vulnerability of the hippocampus to fasting-induced reduction of O-GlcNAcylation and elevation of tau phosphorylation than the cerebral cortex were also seen previously (Li et al., 2006). This differentiative vulnerability of tau hyperphosphorylation is fully consistent to the fact that the hippocampus is more vulnerable to neurodegeneration than the cerebral cortex in AD, further supporting the role of CCH in the development of AD. Taken together, these findings suggest that CCH leads to decreased O-GlcNAcylation and then hyperphosphorylation of tau. Abnormally hyperphosphorylated tau might also contribute to the increased anxiety observed in the CCH mice since a link between anxiety-related behavior and tau hyperphosphorylation was reported previously (Pristera et al., 2013).

It is surprising that the increased tau phosphorylation was also seen at the contralateral side, where O-GlcNAcylation was not decreased, in the UCCAO mice. These observations suggest that an additional mechanism might be involved in tau hyperphosphorylation at the contralateral side of the UCCAO mice. Alternatively, it is possible that the mice had produced sufficient cross circulation between two hemispheres during the 2.5 month period after UCCAO leading to a significant reduction of the perfusion in the contralateral hemisphere due to blood steal, although this phenomenon was not seen 28 days after UCCAO (Kitagawa et al., 2005). This possibility is supported by our observations of increased GLUT1 level in the contralateral cortex and a clear tendency of increased GLUT3 level in the contralateral cortex and hippocampus. Alterations of synaptic proteins and some insulin signaling proteins, as well as slight increase in the number of degenerative neurons, were also seen in the contralateral side of the mouse brains after UCCAO.

Synapses are the structural basis of neural connection, neural plasticity and cognitive function. The clinical symptoms of AD correlate highly to the synaptic loss in the brain (Scheff et al., 2011). Elevated levels of synaptic proteins, such as of synaptophysin, syntaxin, and SNAP-25, are observed in human brains with Braak stages 3 and 4 (Mukaetova-Ladinska et al., 2000), whereas loss of synaptic proteins is a relatively late phenomenon in AD brain, occurring well after the onset of clinically detectable dementia and the appearance of Aβ plaques and NTFs (Mukaetova-Ladinska et al., 2000). The present study also showed altered levels of synaptic proteins, including increased pre-synaptic protein synapsin and post-synaptic protein PSD95, as well as decreased pre-synaptic protein synaptophysin, in the cerebral cortex and/or the hippocampus of the CCH mice 2.5 months after UCCAO. These alterations of the synaptic proteins suggest alterations of synaptic vesicle integrity and functions and might underlie the molecular basis of the mild cognitive deficits of the CCH mice. Decreased level of synaptophysin and alterations of synaptic ultrastructure in the CA1 area of the hippocampus have been observed in rat brains after chronic cerebrovascular hypoperfusion (Wang et al., 2010).

Recent studies have established that brain insulin signaling plays an important role in the regulation of neuronal activity and neural plasticity (van der Heide et al., 2006; Reagan, 2007). Dysregulation of brain insulin signaling has been reported in AD (Frolich et al., 1999; Rivera et al., 2005; Steen et al., 2005; Liu et al., 2011). Various changes of the insulin signaling pathway are seen in different models of neurodegenerative disorders (Pedersen and Flynn, 2004; Chen et al., 2012, 2013). The different changes of insulin signaling in AD and in these models might represent a diversity of insults, which might ultimately contribute to cognitive impairment. In the present study, majority of the alterations of the insulin signaling pathway was increases rather than decreases, which might represent possible compensation in response to CCH. These compensative changes resulted in a marked increase in the inhibitory Ser9 phosphorylation of GSK-3β and thus a marked inhibition of its kinase activity. However, the GSK-3β phosphorylation sites of tau were hyperphosphorylated rather than hypophosphorylated in the CCH mouse brains, suggesting that other opposing factors, such as reduction of O-GlcNAcylation and probably activation of some other tau kinases, had counteracted the effect of GSK-3β inhibition on tau phosphorylation after CCH.

No detectable ischemia or cell death were observed previously in the brains of 3–4 months old mice after UCCAO (Kitagawa et al., 2005; Pimentel-Coelho et al., 2013). In the present study, however, we found degenerative neurons by using Fluoro-Jade staining in the brains, especially in the granule neurons of the dentate gyrus, of the brains in 7–8.5 months old CCH mice. Furthermore, these degenerative neurons were filled with hyperphosphorylated tau, suggesting that neurodegeneration might be attributed to tau phosphorylation in the CCH mouse brains. As the granule neurons of the dentate gyrus receive inputs from the entorhinal cortex through the perforant pathway and send signals to the CA3 pyramidal neurons through the mossy fiber pathway, which makes the critical pathways for memory processing and recording, neuronal degeneration in these areas, together with the synaptic deficits, might underlie the basis of mild memory impairments observed in the CCH mice in the present study.

In conclusion, the present study shows that CCH induced by UCCAO decreased the protein O-GlcNAcylation, increased tau phosphorylation, dysregulated synaptic proteins and brain insulin signaling, and caused mild cognitive impairment. These studies provide experimental evidence showing the possible mechanisms by which CCH can contribute to cognitive impairment and AD probably through reduction of protein O-GlcNAcylation, hyperphosphorylation of tau, dysregulated synaptic plasticity and neural degeneration in the brain. These findings also provide a possible mechanistic linkage between AD and VaD.

## AUTHOR CONTRIBUTIONS

Conception of the research: Cheng-Xin Gong, Yang Zhao, Jin-hua Gu, Qun Liu, Khalid Iqbal, and Fei Liu. Performing experiments: Yang Zhao, Jin-hua Gu, and Chun-ling Dai. Analyses and interpretation of results: Yang Zhao, Jin-hua Gu, Chun-ling Dai, Qun Liu, Khalid Iqbal, Fei Liu, and Cheng-Xin Gong. Drafting of the manuscript: Yang Zhao. Critical revision of the manuscript: Cheng-Xin Gong, Yang Zhao, Jin-hua Gu, Cheng-Xin Dai, Qun Liu, Khalid Iqbal, and Fei Liu.

## Conflict of Interest Statement

The authors declare that the research was conducted in the absence of any commercial or financial relationships that could be construed as a potential conflict of interest.
